# Early effects of COVID-19 on maternal and child health service disruption in Mozambique

**DOI:** 10.3389/fpubh.2023.1075691

**Published:** 2023-04-17

**Authors:** Orvalho Augusto, Timothy Roberton, Quinhas Fernandes, Sérgio Chicumbe, Ivan Manhiça, Stélio Tembe, Bradley H. Wagenaar, Laura Anselmi, Jon Wakefield, Kenneth Sherr

**Affiliations:** ^1^Department of Global Health, School of Public Health, University of Washington, Seattle, WA, United States; ^2^Department of Community Health, Faculdade de Medicina, Eduardo Mondlane University, Maputo, Mozambique; ^3^Johns Hopkins Bloomberg School of Public Health, Johns Hopkins University, Baltimore, MD, United States; ^4^National Directorate of Public Health, Ministry of Health, Maputo, Mozambique; ^5^National Institute of Health, Ministry of Health, Marracuene, Mozambique; ^6^Direcção Provincial de Inhambane, Inhambane, Mozambique; ^7^Department of Epidemiology, School of Public Health, University of Washington, Seattle, WA, United States; ^8^Health Organization, Policy and Economics, Centre for Primary Care and Health Services Research, University of Manchester, Manchester, United Kingdom; ^9^Department of Biostatistics, School of Public Health, University of Washington, Seattle, WA, United States; ^10^Department of Statistics, University of Washington, Seattle, WA, United States; ^11^Department of Industrial and Systems Engineering, University of Washington, Seattle, WA, United States

**Keywords:** COVID-19, MCH, Mozambique, interrupted time-series analysis, seasonality, LMIC, PALOP

## Abstract

This article is part of the Research Topic ‘Health Systems Recovery in the Context of COVID-19 and Protracted Conflict'

**Introduction:**

After the World Health Organization declared COVID-19 a pandemic, more than 184 million cases and 4 million deaths had been recorded worldwide by July 2021. These are likely to be underestimates and do not distinguish between direct and indirect deaths resulting from disruptions in health care services. The purpose of our research was to assess the early impact of COVID-19 in 2020 and early 2021 on maternal and child healthcare service delivery at the district level in Mozambique using routine health information system data, and estimate associated excess maternal and child deaths.

**Methods:**

Using data from Mozambique's routine health information system (SISMA, Sistema de Informação em Saúde para Monitoria e Avaliação), we conducted a time-series analysis to assess changes in nine selected indicators representing the continuum of maternal and child health care service provision in 159 districts in Mozambique. The dataset was extracted as counts of services provided from January 2017 to March 2021. Descriptive statistics were used for district comparisons, and district-specific time-series plots were produced. We used absolute differences or ratios for comparisons between observed data and modeled predictions as a measure of the magnitude of loss in service provision. Mortality estimates were performed using the Lives Saved Tool (LiST).

**Results:**

All maternal and child health care service indicators that we assessed demonstrated service delivery disruptions (below 10% of the expected counts), with the number of new users of family planing and malaria treatment with Coartem (number of children under five treated) experiencing the largest disruptions. Immediate losses were observed in April 2020 for all indicators, with the exception of treatment of malaria with Coartem. The number of excess deaths estimated in 2020 due to loss of health service delivery were 11,337 (12.8%) children under five, 5,705 (11.3%) neonates, and 387 (7.6%) mothers.

**Conclusion:**

Findings from our study support existing research showing the negative impact of COVID-19 on maternal and child health services utilization in sub-Saharan Africa. This study offers subnational and granular estimates of service loss that can be useful for health system recovery planning. To our knowledge, it is the first study on the early impacts of COVID-19 on maternal and child health care service utilization conducted in an African Portuguese-speaking country.

## 1. Introduction

As of July 6, 2021, 15 months after the World Health Organization (WHO) declared the severe acute respiratory syndrome coronavirus 2 (SARS-CoV-2) a global pandemic, there were more than 184 million cases, resulting in 4 million deaths worldwide (1). By December 31, 2020, 3 million deaths had been recorded worldwide, corresponding to 1.2 million excess deaths in 2020. However, these are likely to be underestimates (2). Moreover, it is challenging to distinguish between direct and indirect deaths as a result of potential service disruption due to the COVID-19 pandemic. Early forecasts from May 2020 estimated a 9.8–44.7% increase in under-five child deaths and an 8.3–38.6% increase in maternal deaths per month due to service disruption of several maternal and child interventions in 118 low-income and middle-income countries (3).

Mozambique reported its first case of COVID-19 on March 22, 2020, with 80,888 cases reported resulting in 912 deaths as of July 6, 2021 (4). Two major outbreak waves were observed between March 2020 and April 2021, and a third wave occurred between June and September 2021. The third wave was dominated by the delta variant, with many reported cases and a higher fatality rate; however, by that time the country had vaccinated health care workers and had begun to vaccinate other major risk groups (e.g., older adults over 60 years) and had increased capacity for diagnosis and management of severe COVID-19 cases.

Early in the epidemic Mozambique established a scientific committee to guide and counsel the government's COVID-19 response, mounted a surveillance system for SARS-CoV-2 infection, and enacted a 6-month state of emergency beginning April 1, 2020 that included a range of measures to limit the spread of infections including (a) actions for personal protection (e.g., promotion of hand washing and enforcement of face mask wearing), (b) social measures and prohibition of public gatherings (social distancing; closing schools, churches, beaches, and gyms; reducing the number of workers; and instituting curfews), (c) travel restrictions, (d) environmental measures (e.g., disinfection of surfaces frequently touched) (5, 6). These actions—combined with a general public fear of contracting SARS-CoV-2 and misinformation about the source and treatment of COVID-19—potentially led to lower rates of health service utilization (3, 7).

Health information systems are core components of functional health systems as they ensure the production, analysis, dissemination and use of reliable and timely information on health service utilization, health determinants and health status (8, 9). Routine health information systems (RHIS) provide regular, repeated data that is multilevel (including data from primary care facilities to differentiated care) that can be used to establish utilization patterns and to detect deviations from these patterns that can be used to describe the magnitude and duration of disruptions to health service utilization. Such assessments are essential to plan for health service recovery, including monitoring the effects of corrective interventions (9).

The purpose of our research was to assess the early impact (2020 and early 2021) of COVID-19 on utilization of maternal and child health services at the district level in Mozambique based on routine health information system data. In addition, we aimed to estimate excess maternal and child mortality due to these losses.

## 2. Materials and methods

### 2.1. Study design

Using data from the country's routine health information system (SISMA, *Sistema de Informação em Saúde para Monitoria e Avaliação*), we conducted a time-series analysis to assess changes in selected indicators of maternal and child health care service provision in all districts of Mozambique in 2020 to March 2021, before widespread detection of the delta variant.

Routine health data are collected primarily *via* paper registers at health facilities and aggregated in monthly reports at the district level. These reports are then entered into SISMA. The dataset used for our analysis was extracted as counts of services provided from January 2017 to March 2021 for each of Mozambique's 159 districts. Since October 2019, there has been military conflict affecting vast areas of Cabo Delgado Province, leading to unprecedented population loss and displacement, as well as the destruction and closure of health facilities. We therefore decided to remove data from the Cabo Delgado Province from our analysis (Box 1).

Box 1 **Cabo Delgado Province under military attack (10).**

Since October 2017, the northern province of Cabo Delgado has been under armed nonstate group attacks. During 2020 there was an escalation of the attacks with brutal attacks toward civilians, setting fire to homes, shops, schools, and religious and government buildings, and forcing people to flee into the bush and neighboring villages and provinces. Between April 2020 and April 2021, the number of internally displaced people (IDP) increased from 172,000 to 732,000. Around 72% of the displaced live with host communities, while 28% are in IDP camps.

### 2.2. Study setting and overview of Mozambique and its health system

**Figure 1:**
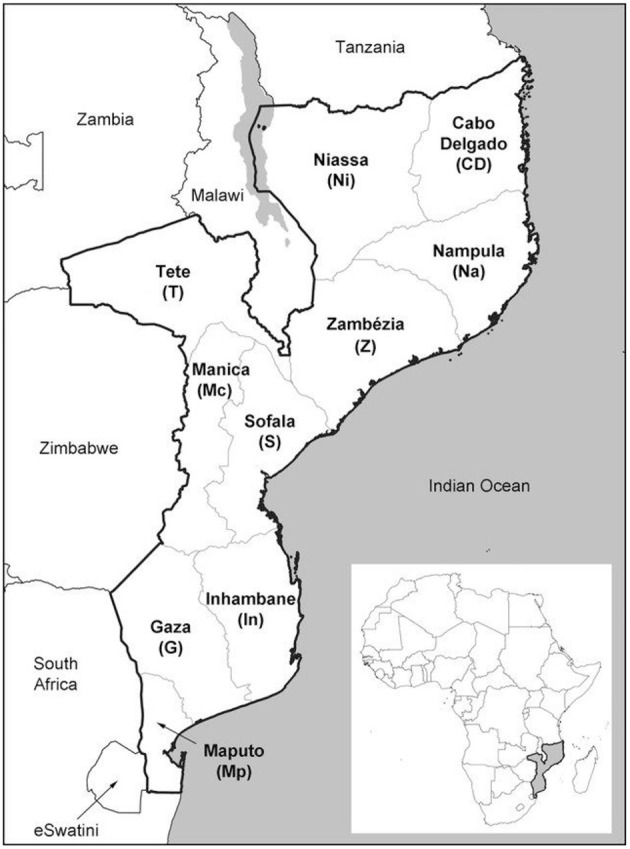
Map of Mozambique with administrative divisions (provinces). Maputo City (not in the illustration) is contained within Maputo Province. Source: Darbyshire et al. (11).

Mozambique is in southeastern Africa (Figure 1), with a surface area of 801,590 km^2^ and 2,700 km of coastal line. It is crossed from west to east by large rivers, isolating parts of the country during the rainy season (between October and March), a situation that is worsening with climate change. Administratively, the country is divided into 11 provinces. Apart from Maputo City and Maputo Province, each province is divided into ~15 districts, with a total of 159 districts in the country. The 2017 National Census recorded 26,899,105 inhabitants, with a median age of 16.6 years, life expectancy at birth of 53.7 years, and an estimated 2.8% yearly population growth rate (12). Children under five and women of reproductive age (ages 15–49) corresponded to 34.6 and 23.8% of the population, respectively. Overall Mozambique has a low population density, with 34.1 inhabitants per km^2^, except for Maputo City where the density is 3,107.1 inhabitants per km^2^ (Table 1). In 2019 the infant mortality ratio (IMR) was estimated at 51.0 deaths per 1,000 live births—among the 20 highest globally even after an average annual decrease of 2.6% since 1990 (13). The maternal mortality ratio was estimated at 289 deaths per 100,000 live births in 2017, the 3rd worst figure among the 16 nations in SADC (Southern African Development Community) region and one of the top 20 high in sub-Saharan Africa (14). Mozambique is a low-income country with a gross national income per capita in 2019 of US$504 (15). Mozambique ranks 181 out of 189 countries assessed in the Human Development Index (16).

**Table 1:** Mozambique demographic characteristics by province.

**Province**	**Population**	Women (%)^*^	Women of reproductive age (%)^*^	Children under 5 (%)^*^	**Population density (inhabitants/km^2^)**	**Crude birth rate**	**Number of districts**
Cabo Delgado^†^	2,267,715	51.6	22.8	35.5	29.0	39.6	17
Gaza	1,388,039	54.8	24.7	28.1	18.4	31.6	14
Inhambane	1,454,804	54.3	24.5	26.4	21.1	28.8	14
Manica	1,851,931	52.1	23.5	36.0	29.6	44.0	12
Maputo City	1,080,277	51.7	29.0	20.8	3,107.1	24.5	1^‡^
Maputo Province	1,908,078	52.2	27.7	24.6	81.8	30.3	8
Nampula	5,483,382	51.5	22.8	38.6	69.9	38.2	23
Niassa	1,713,751	51.4	22.2	39.7	13.2	41.9	19
Sofala	2,196,845	51.7	23.9	33.5	32.3	40.7	13
Tete	2,551,826	51.2	22.9	35.6	25.3	38.0	15
Zambézia	5,002,457	52.1	23.1	38.6	48.4	43.3	23
Total	26,899,105	52.0	23.8	34.6	34.1	37.9	159

^*^Proportions over total population.

^†^Cabo Delgado was excluded from analysis due to insecurity and military conflict affecting the region.

^‡^Maputo City has seven municipal districts. Municipal districts are not equivalent to districts as in other provinces.

The national health system provides nearly all health care services throughout the country, whereas private sector facilities are available primarily in Maputo City and a few provincial capitals. The national health system is divided into four levels of progressively more complex care. (1) The primary level (health centers or posts) provides primary care, including basic maternal and child health services in most facilities. (2) Secondary level facilities (rural, district, or general hospitals) are located at the district level, and serve as referral facilities for primary level facilities (note that some secondary level hospitals may include surgical services such as cesarean sections). (3) The tertiary level consists of hospitals located in provincial capitals, acting as referral facilities for the province. (4) The quaternary level (central hospitals) serve as regional (North, Center, or South) referral facilities (17).

### 2.3. Data sources and processing

Monthly data on health service provision at the district level were extracted from SISMA in April 2021 following a request from the National Directorate of Public Health (DNSP, Direcção Nacional de Saúde Pública) to assess the impact of COVID-19 on maternal and child health routine indicators. The population data (total women of reproductive age and children under five) were extracted from Government of Mozambique projections using the 2017 National Census (7).

A set of nine indicators were selected based on data availability, completeness, and whether the indicators could be used in the Lives Saved Tool (LiST) (18) for mortality estimation. The indicators included are: (1) number of women who attended first antenatal care visits; (2) number of institutional deliveries; (3) number of visits for measles vaccination; (4) number of visits for third dose of DPTH (diphteria, pertussis, tetanus toxoid combined with hepatitis B) vaccination; (5) number of postnatal care visits within 48 h after delivery; (6) number of postnatal care visits within three to seven days after delivery; (7) number of visits for treatment of malaria with Coartem for children under five; (8) number of first well-child visits; and (9) number of new family planning users. Data completeness was assessed as the proportion of district-months without missing information. Indicators missing fewer than 10% district-months were kept for analysis (Table 2) with two exceptions—treatment of malaria with Coartem and first well-child visit. These indicators were retained as measures of child health care utilization are required to estimate mortality using LiST. The team agreed that the data were sufficiently reliable to use for mortality estimation, and that while there would be greater uncertainty in LiST findings, they would still provide important guidance.

**Table 2:** Selected maternal and child health indicators for analysis between January 2017 and December 2019 (district-month counts).

**Indicator**	**Number of observations**	**Missing (%)**	**Minimum**	**Maximum**	**Mean**	**SD**	**CV**	Relative annual growth (%)^*^
Antenatal care (first visit)	4,895	5 (0.1)	6	5,577	854	656	0.77	4.5
Institutional delivery	4,899	1 (0.0)	5	3,336	571	429	0.75	5.4
Measles vaccination	4,895	5 (0.1)	3	7,563	604	488	0.81	4.8
DPTH3 vaccination	4,895	5 (0.1)	4	8,549	604	479	0.79	6.1
Postnatal care visit (within 48 h)	4,897	3 (0.1)	5	2,248	507	375	0.74	8.2
Postnatal care visit (within 3–7 days)	4,545	355 (7.8)	1	1,138	87	107	1.23	−3.3
Treatment of malaria with Coartem (children under 5)^†^	1,476	204 (13.8)	0	9,270	1,322	1,347	1.02	−1.6
First well-child visit^†^	1,502	178 (11.9.6)	0	7,436	1,286	1,790	1.39	−4.6
Family planning (new users)^¶^	3,219	1 (0.0)	19	10,350	1,503	1,449	0.96	0.7

SD, standard deviation; CV, coefficient of variation (the ratio of the SD to the mean); DPTH3, third dose of diphtheria, pertussis, tetanus, and haemophilus influenza vaccine.

^*^Estimated from a generalized linear model regression with family quasipoisson and log-link for descriptive purposes.

^†^No data available on SISMA before January 2019.

^¶^No data available on SISMA before January 2018.

### 2.4. Statistical analysis

The outcome of our analysis were the service utilization counts for each indicator, aggregated at the district level. We conducted an exploratory analysis to assess data completeness, identify potential outliers, and determine the model parametrization type. Descriptive statistics (mean, standard deviation, coefficient of variation, median, first and third quartile, and proportion) were used for district comparisons, and district-specific time-series plots were produced to select the final model parametrization. Given higher unpredictability in trends in 2020 and 2021, we modeled linear trends using data prior to 2020. We then used the model predictions for each month in 2020 and the first quarter of 2021. We compared the observed counts with the expected (predicted) counts as absolute differences and ratios to provide a measure of the magnitude of loss in service provision for maternal and child health services as a result of COVID-19.

To model the data prior to 2020, we used a hierarchical negative binomial regression model with district- and province-level random effects for intercepts and district-level slopes. A negative binomial model was chosen to accommodate count outcome data with potentially greater variability than a common Poisson model. The hierarchical model addresses that districts are nested within provinces (i.e., at the highest level is the between-province variability); the multiple observations per district (i.e., between districts variability); and month-to-month within-district variability. In addition, we account for annual seasonality variability. The model has the following parametrization:


$$\begin{array}{l} log(count_{dt})=(\beta_{0}+b_{0}{}^{*d}+b_{0}{}^{*p})+(\beta_{1}+b_{1}{}^{*d})\cdot t\\ +\sum_{m=2}^{12}\delta_{m}\cdot I(Month_{t}=m)+1\cdot [log(Population_{dt})]\; \end{array}$$

Where *count* is the count of service delivery from a district *d* at time *t*. The variable and subscript *t* variable *time* index time in months from January 2017 through December 2019. The parameters of interest are the β's and b's. The β_0_ represents in log-scale the overall district average counts in January 2017, whereas the $b_{0}*d$ and $b_{0}*p$ are deviations of a particular district d and province p, respectively. The β_1_ represents in log-scale the monthly average increase with $b_{1}*d$ a particular district deviation. The summation with δ and I (Month_t_ = m) are dummy indicator variables to capture monthly seasonal deviations from a January month. To account for the differences in district populations, district population is introduced as an offset (an independent variable in the model without a coefficient estimated) in the model. All regression models were estimated through the Bayesian framework using Stan programming language in the brms package (19) of R version 3.6.3 (20). The software default priors (uninformative and diffuse priors) were found to be appropriate for this analysis after the number of iterations was increased to 20,000 (5,000 per chain), and a thinning interval of 5 and 1,000 observations in the burn-up period. From the posterior distribution, 10,000 realizations of the sets of parameters were obtained and used to estimate the absolute and relative loss of service delivery for each month in 2020 and the first quarter of 2021.

Mortality estimates were performed using the LiST (18), which is a mathematical model that uses community-level maternal and child health service data as inputs to estimate maternal and child mortality. We used coverage estimates from the 2015 Immunization, Malaria and HIV/AIDS Indicator Survey (21) and the 2011 Demographic and Health Survey (22) as inputs for service coverage data. Changes in health service utilization were estimated using RHIS data aggregated at the district level, which is assumed to reflect the experience of district populations given the high utilization of maternal and child health services through the public sector, as well as infrequent population migration in Mozambique.

### 2.5. Ethical considerations

For this study we used district-level aggregated data, with approval from the Ministry of Health. We extracted data from the routine health information system (SISMA). Because routine data do not contain personal identifiers, ethical approval was deemed unnecessary. However, the National Directorate of Public Health approved the use of the data.

## 3. Results

### 3.1. Trends before 2020

From January 2017 through December 2019, with the exception of Cabo Delgado Province, 140 districts reported data. Table 2 describes the selected maternal and child health indicators prior to January 2020. On average, a typical district per month reported 854 new first antenatal care consults, 571 institutional deliveries, 507 postnatal visits within 48 h after delivery, 87 postnatal visits within 3–7 days after delivery, and 604 visits for a third dose of the combined diphtheria, pertussis, tetanus, and haemophilus influenza (DPTH3) vaccine. However, there is a large district-level variation with the coefficients of variation (CV) being at least 75%, i.e., for each indicator the standard deviation is above three quarters of its mean, and reaching above 100% among the monthly counts of post-antenatal care visits, malaria treatment with Coartem and first well-child visits. Antenatal care visits, institutional deliveries, measles and DPTH3 doses, and postnatal care visits within 48 h of delivery saw a relative annual growth of about 5%, whereas the other indicators saw a smaller magnitude year descending trend. The number of family planning new users remained stable in the years before COVID-19.

Table 3 shows the regression coefficients for service provision counts per 1,000 inhabitants (except for the first well-child visits, which is in counts per 100,000 children) between January 2017 and December 2019, accounting for population size and seasonality. Except for postnatal care visits (within 3–7 days) and first well-child visits, all indicators had a relative growth of 2–7% per year, apart from treatment of malaria with Coartem for children under age five, which reached 35% increase per year. The standard deviation of the random effects illustrates heterogeneity between districts (district random intercept) and provinces (province random intercept) as well as the trajectory of the indicators over time (district random slope).

**Table 3:** Mixed-effect negative binomial regression coefficients for selected indicators, January 2017 to December 2019.

**Indicator**	**Intercept (per 1,000 inhabitants)**	**Time (year)**	σ_district_intercept_^*^	σ_district_slope_^*^	σ_province_intercept_^*^
Antenatal care (first visit)	7.92 (6.56–9.60)	1.02 (1.01–1.03)	0.203 (0.178; 0.233)	0.045 (0.038; 0.053)	0.283 (0.165; 0.512)
Institutional delivery	5.42 (4.57–6.40)	1.03 (1.02–1.04)	0.245 (0.215; 0.280)	0.049 (0.042; 0.057)	0.244 (0.133; 0.435)
Measles vaccination	5.89 (5.09–6.81)	1.02 (1.00–1.03)	0.199 (0.172; 0.230)	0.053 (0.043; 0.064)	0.196 (0.104; 0.353)
DPTH3 vaccination	5.87 (5.04–6.82)	1.02 (1.01–1.03)	0.180 (0.157; 0.207)	0.036 (0.028; 0.045)	0.216 (0.121; 0.381)
Postnatal care visit (within 48 h)	4.64 (3.69–5.78)	1.05 (1.03–1.06)	0.299 (0.261; 0.342)	0.074 (0.064; 0.086)	0.334 (0.188; 0.587)
Postnatal care visit (within 3–7 days)	0.83 (0.69–0.99)	0.88 (0.83–0.94)	0.641 (0.556; 0.736)	0.312 (0.269; 0.363)	0.158 (0.007; 0.440)
Treatment of malaria with Coartem (children under 5)^†^	8.13 (2.83–25.37)	1.35 (1.21–1.49)	0.635 (0.539; 0.744)	0.277 (0.225; 0.335)	0.299 (0.069; 0.625)
First well-child visit^†^	18.82 (14.32–25.25)	0.89 (0.82–0.96)	0.545 (0.394; 0.689)	0.219 (0.152; 0.288)	1.656 (1.019; 2.775)
Family planning (new user)^¶^	14.87 (12.00–18.44)	1.04 (1.02–1.07)	0.369 (0.273; 0.468)	0.139 (0.096; 0.181)	0.398 (0.214; 0.715)

^*^Standard deviations of the random effects are on the log scale.

^†^Data available on SISMA since January 2019. First well-child visits are measured per 100,000 children under five.

^¶^Data available on SISMA since January 2018.

The intercept indicates the average rate (counts per 1,000 inhabitants) on January 2017. The time coefficient indicates the increasing factor per year (e.g., the antenatal care visits rate increases 1.02 times in a year). The sigmas are an indication of the source of variability for the intercept and slope.

### 3.2. Service disruptions

Table 4 shows the observed and expected service counts in 2020 and for the first quarter of 2021. We estimated relative losses for all service provision indicators at the national level. Our findings show that all services experienced losses, but the services most affected were family planning (number of new users) and malaria treatment with Coartem (number of children under five treated), which showed relative losses of more than a quarter (29.87 and 29.62%, respectively) compared with what was expected. Other services sustained losses of < 10% of what was expected in 2020, with substantial increases in losses during the first quarter of 2021.

**Table 4:** Mozambique observed and expected service losses by indicator, 2020 and first quarter of 2021.

**Indicator**	**2020**				**First Quarter 2021**				**Overall**			
	**Observed**	**Expected**	**Difference**	**Percentage loss (%)** ^*^	**Observed**	**Expected**	**Difference**	**Difference (%)** ^*^	**Observed**	**Expected**	**Difference**	**Percentage loss (%)** ^*^
Antenatal care (first visit)	1,752,157	1,787,674	−35,517	−1.99	446,890	475,221	−28,331	−5.96	2,199,047	2,262,894	−63,847	−2.82
Institutional delivery	1,169,966	1,223,790	−53,823	−4.40	298,712	312,064	−13,351	−4.28	1,468,679	1,535,853	−67,174	−4.37
Measles vaccination	1,164,151	1,254,650	−90,498	−7.21	268,441	301,688	−33,248	−11.02	1,432,592	1,556,338	−123,746	−7.95
DPTH3 vaccination	1,164,712	1,274,312	−109,599	−8.60	224,389	309,538	−85,149	−27.51	1,389,102	1,583,850	−194,748	−12.30
Postnatal care visit (within 48 h)	1,053,793	1,149,645	−95,852	−8.34	266,587	294,773	−28,186	−9.56	1,320,381	1,444,418	−124,037	−8.59
Postnatal care visit (within 3–7 days)	159,760	160,383	−623	−0.39	38,513	43,899	−5,387	−12.27	198,273	204,283	−6,010	−2.94
Treatment of malaria with Coartem (children under 5)	2,815,039	3,685,427	−870,389	−23.62	645,443	1,231,383	−585,940	−47.58	3,460,482	4,916,811	−1,456,329	−29.62
First well-child visit	2,145,752	2,334,514	−188,763	−8.09	–	559,308	–	–	–	2,893,823	–	–
Family planning (new user)	2,229,723	3,240,669	−1,010,946	−31.20	533,932	700,080	−166,148	−23.73	2,763,655	3,940,749	−1,177,094	−29.87

The observed counts in the table are slightly different from the raw data, because here we computed first the average (per indicator) then multiplied by 159 (total number of districts) to account for districts with missing information.

The expected counts are estimates from the model for each parameter.

^*^The percentage loss is computed as 100 × (observed – expected)/expected.

The monthly ratio of observed counts to expected counts reveals immediate losses in April 2020 (Table 5) for nearly all indicators, with ratios below 0.90. Most of those indicators had sustained losses (ratios below 0.95) for more than 3 months, except for first antenatal care visits and first well-child visits. However, these patterns varied slightly by province (Supplementary Figures S1–S9). The provinces of Manica, Maputo City, Nampula, and Sofala experienced the most severe losses in the number of family planning new users.

**Table 5:** National-level relative reduction in service counts by indicator and month, 2020 and first quarter of 2021.

**Indicator**	**2020**												**2021**		
	**Jan**	**Feb**	**Mar**	**Apr**	**May**	**Jun**	**Jul**	**Aug**	**Sep**	**Oct**	**Nov**	**Dec**	**Jan**	**Feb**	**Mar**
Antenatal care (first visit)	0.96 (0.88–1.04)	0.99 (0.91–1.08)	1.03 (0.95–1.13)	0.87 (0.79–0.95)	0.99 (0.90–1.09)	0.95 (0.86–1.04)	1.00 (0.90–1.10)	1.08 (0.97–1.19)	0.98 (0.88–1.08)	1.08 (0.96–1.20)	1.01 (0.90–1.13)	0.90 (0.80–1.01)	1.07 (0.95–1.20)	0.96 (0.85–1.09)	1.01 (0.89–1.14)
Institutional delivery	0.95 (0.73–1.22)	1.04 (0.79–1.34)	1.02 (0.77–1.34)	0.78 (0.58–1.03)	0.66 (0.49–0.90)	0.82 (0.59–1.11)	0.91 (0.66–1.25)	0.98 (0.70–1.37)	1.07 (0.75–1.50)	1.11 (0.77–1.58)	1.07 (0.73–1.54)	1.05 (0.71–1.54)			
Measles vaccination	0.99 (0.90–1.09)	1.01 (0.91–1.11)	1.02 (0.92–1.13)	0.84 (0.75–0.93)	0.92 (0.82–1.02)	0.85 (0.76–0.95)	0.94 (0.83–1.05)	0.96 (0.85–1.08)	0.88 (0.77–0.99)	0.96 (0.84–1.08)	0.87 (0.77–1.00)	0.88 (0.77–1.00)	0.92 (0.80–1.05)	0.81 (0.70–0.93)	0.83 (0.72–0.96)
DPTH3 vaccination	0.97 (0.86–1.09)	1.04 (0.92–1.17)	1.08 (0.95–1.22)	0.82 (0.72–0.93)	0.90 (0.79–1.03)	0.87 (0.76–1.00)	0.91 (0.79–1.05)	1.00 (0.86–1.15)	0.89 (0.76–1.03)	0.94 (0.81–1.10)	0.86 (0.73–1.01)	0.96 (0.81–1.13)	1.03 (0.87–1.21)	0.93 (0.78–1.11)	0.97 (0.81–1.15)
Postnatal care (48 h)	0.92 (0.73–1.14	0.99 (0.78–1.24)	0.99 (0.77–1.26)	0.80 (0.62–1.02)	0.72 (0.55–0.92)	0.72 (0.55–0.94)	0.76 (0.57–0.99)	0.83 (0.62–1.10)	0.71 (0.53–0.94)	0.75 (0.55–1.01)	0.49 (0.36–0.67)	0.76 (0.55–1.04)	0.93 (0.66–1.28)	0.89 (0.63–1.24)	0.85 (0.60–1.20)
Postnatal care (within 3–7 days)	0.99 (0.92–1.05)	0.99 (0.92–1.06)	1.00 (0.93–1.08)	0.92 (0.85–0.99)	0.97 (0.90–1.05)	0.94 (0.87–1.02)	0.95 (0.87–1.04)	0.94 (0.86–1.03)	0.95 (0.87–1.05)	0.96 (0.87–1.06)	0.96 (0.87–1.05)	0.95 (0.86–1.05)	1.04 (0.94–1.16)	1.01 (0.91–1.12)	1.01 (0.91–1.13)
Treatment of malaria with Coartem (children under 5)	1.01 (0.72–1.39)	0.97 (0.68–1.34)	1.02 (0.70–1.46)	0.89 (0.60–1.29)	0.78 (0.52–1.17)	0.76 (0.49–1.15)	0.73 (0.47–1.12)	0.67 (0.42–1.04)	0.62 (0.38–0.99)	0.84 (0.51–1.36)	0.82 (0.49–1.35)	0.63 (0.37–1.07)	0.70 (0.37–1.33)	0.69 (0.38–1.25)	0.79 (0.44–1.38)
First well-child visit	1.04 (0.62–1.71)	1.01 (0.58–1.74)	1.48 (0.80–2.76)	0.84 (0.47–1.48)	1.11 (0.60–2.06)	0.94 (0.49–1.83)	1.27 (0.64–2.55)	1.30 (0.65–2.62)	1.41 (0.67–2.96)	1.10 (0.54–2.22)	2.17 (1.02–4.53)	0.76 (0.35–1.65)			
Family planning (new user)	0.92 (0.73–1.14)	0.99 (0.78–1.24)	0.99 (0.77–1.26)	0.80 (0.62–1.02)	0.72 (0.55–0.92)	0.72 (0.55–0.94)	0.76 (0.57–0.99)	0.83 (0.62–1.10)	0.71 (0.53–0.94)	0.75 (0.55–1.01)	0.49 (0.36–0.67)	0.76 (0.55–1.04)	0.85 (0.60–1.20)	0.89 (0.63–1.24)	0.93 (0.66–1.28)

### 3.3. Mortality impact

Using the LiST for 2020, the number of estimated excess deaths (and relative increase) is 11,337 (12.8%) children under five, 5,705 (11.3%) neonates, and 387 (7.6%) mothers (Table 6), compared to a scenario without COVID-19.

**Table 6:** Mozambique's relative reduction in service counts, by indicator and month for 2020 and first quarter of 2021.

	**Total expected deaths in 2020 with no disruptions (counterfactual)**	**Total estimated deaths in 2020 with observed disruptions**	**Excess deaths in 2020 due to service disruptions**	**Relative increase in mortality due to service disruptions**
Child deaths (0–59 months of age)	88,853	100,190	11,337	12.8%
Neonatal deaths (newborns < 1 month of age)	50,311	56,016	5,705	11.3%
Maternal deaths	5,084	5,471	387	7.6%

## 4. Discussion

To assess the early impacts of COVID-19 on maternal and child health service provision and maternal and child mortality in Mozambique, we analyzed data from the country's routine health information system between January 2020 and March 2021 and compared it with data from before the pandemic. We focused on the early stages of the pandemic when a COVID-19 vaccine was not yet available, there was limited and centralized laboratory capability to diagnose COVID-19, there was little to no clinical experience with a respiratory distress disease outbreak, and before the delta variant emerged as the dominant strain. Moreover, during this period, the government of Mozambique instituted a state of emergency between April and August 2020, then changed to a state of public calamity in September 2020. These restrictions included police-reinforced measures to reduce movement and access to public spaces and services (5). From April 2020 through March 2021 we found evidence of substantial service provision loss in selected indicators across the maternal child health care continuum in Mozambique. These losses are estimated to have contributed to a substantial increase in maternal, neonatal, and child mortality in 2020. Our findings complement findings from a report published in April 2022 indicating an overall negative impact on health services utilization as a result of COVID-19 from March through December 2020 in Mozambique (6). In addition, our study adds to the literature on maternal and child health service utilization losses due to COVID-19 in other sub-Saharan African countries (23, 24), and will contribute to planning for Mozambique's health system recovery. To our knowledge, this is the first study on the early impacts of COVID-19 on maternal and child healthcare service utilization and mortality conducted in one of the six African Portuguese-speaking countries.

The overall magnitude of losses in our selected indicators of maternal child health service provision are similar to what was reported for other sub-Saharan countries (24, 25), except for new users of family planning and the number of children treated with Coartem, which in our analysis reached almost 30% of service count loss. The provision of these two services includes an enormous contribution of the community health workers (CHW) in rural areas. The restrictions due to emergency or public calamity state halted CHW activities, which likely contributed to the observed service disruption. It is notable that despite experiencing losses below 10% of expected service counts, other services had accelerated losses in the first quarter of 2021. These losses represent a major blow to the efforts toward universal health coverage; however, the losses could potentially be worse still if there had not been a well-designed and implemented multi-sectorial response to prevent COVID-19 transmission (5). Still, it could be argued, as well, that some of the measures may have contributed to the losses (26).

We can group the losses in our analysis in three patterns of service loss over the months of 2020 and first quarter of 2021 (Table 5). The first includes indicators that experienced 1 month of loss (below 95% of the expected) or did not change over the course of the year relative to the expected. This is the case with first antenatal care visit counts, which in April 2020 were 13% below the expected. The second pattern includes those outcomes reduced to below 95% of what was expected for between 2 and 4 months. This group includes institutional deliveries and first well-child visits. The third group encompasses indicators that sustained more than 4 months with observed counts below 95% of what was expected. This group included immunization indicators (measles and DPTH3), post-natal visits, malaria treatment with Coartem, and family planning visits. Nevertheless, at the sub-national (province) level, there were substantial variations with Maputo City and Maputo Province showing loss of service provision (or utilization) of malaria treatment with coartem for more than 4 months, a completely different pattern to that of the other provinces. Many factors could concur with this observation. First, on the one hand, in recent years, Maputo Province and Maputo City reached a higher community prevalence of knowledge for malaria prevention and treatment that may contribute to lower malaria incidence compared to previous years. On the other hand, these two provinces were the ones with the highest reinforcement of COVID-19 prevention measures that could have contributed to deterred service utilization.

The first and second pattern of loss indicators, including first antenatal care, institutional deliveries and first well-child visits, were targeted by the National Directorate of Public Health in reaction to earlier service count drops after April 2020. The third group of indicators include services that are sensitive to health system changes like service discontinuation to provide COVID-19 treatment or health facility closure, insufficient personal protective equipment available for health care providers, and interruptions in the supply of medical equipment and health products, which likely contributed to their prolonged decrease. Notably, family planning services were the most affected likely due their reliance on community health workers.

Although our estimates of mortality due to service provision loss (an increase of under-5 child mortality by 11% and maternal mortality by 8%) are small relative to other estimates for Mozambique (3, 27), this increased mortality is concerning as it rolls back the hard-fought gains Mozambique has achieved in reducing maternal and child mortality.

Our data analysis is based on selected indicators from the Ministry of Health's RHIS combined with population size estimates at the district level. Due to high levels of missingness (above 40% of the expected observations), a small number of indicators were included in our analysis. This study supports the need for further investments in RHISs as an essential health system building block that supports health system resilience by providing timely monitoring and feedback to health system authorities, and thus are a core element of health service recovery planning (9).

During the COVID-19 pandemic new health information tools were introduced to support the response to the evolving health provision needs (28). However, these changes were largely vertically designed and implemented, and did not integrate into the routine health information system (29). Efforts to transform these vertical systems into broader RHIS improvements should be prioritized in the efforts to restore routine health services. Lessons from this experiment can help identify future opportunities, such as a digital transition of RHIS that may improve responsiveness of the health system in real-time.

### 4.1. Strengths and limitations

We consider the use of RHIS data at the national level over time to be a strength of this study and to provide a model for similar analyses using the RHIS for policy evaluation (30). We used RHIS data which was readily available for 24 months before the COVID-19 pandemic and for the first 15 months of COVID-19. This allowed us to estimate the expected counts for each indicator if COVID-19 didn't happen, an essential step to compare with during COVID-19 service provision counts. Although we could not include Cabo Delgado province, our data is representative of Mozambique.

Of the 11 Mozambican provinces, we could not include Cabo Delgado province because of the military insurgency affecting large areas of the province with public service unavailability, massive population migration, including health providers, and destruction of health facilities and infrastructure. This situation led to an inability to record data into RHIS and to properly assess, monitor and plan the health service provision in Cabo Delgado. This province has a damage beyond service disruption due COVID-19. For Cabo Delgado there is first need to restore peace and then a reconstruction of facilities and restoration of the health system. While the war continues efforts to restore the relief from the humanitarian crisis should be prioritized.

Our estimates are based on the assumption that the magnitude of relative reduction in health facility service utilization represents changes experienced at the district level. While RHIS data do not include services provided through the private sector, and there could be unmeasured changes in population size due to migration patterns, we are confident that our district-level estimates are sound given the lack of observed population migration (particularly under COVID-19 restrictions) and the lack of utilization of private health facilities outside of Maputo City. Second, we cannot provide uncertainty estimates from this model.

We chose a limited number of indicators along the maternal and child health care continuum based on data completeness and history of data collection. Therefore, this dataset might not fully capture service delivery disruptions [for example, malnutrition indicators may have worsened, as was documented during the early pandemic in Eastern and South Africa (31)]. In addition, our analysis was based on routine data from health facilities, which may not capture service utilization at the community level.

## 5. Conclusions

This study provides evidence of the negative impacts of COVID-19 on selected maternal child health service provision in Mozambique during 2020 and the first quarter of 2021. In addition, it estimates the number of children and mothers who died from such service losses. In doing so, it is another example of how RHIS can be used to quickly assess and inform a health system for better action.

These findings have important implications both within Mozambique and for the larger global health community. Plans and guidance for health system recovery can rely on data generated through routine health information system which calls for efforts to be directed to prevent parallel data collection systems. Furthermore, future epidemic responses should consider the essential role that community health workers provide in the health sector (such as in the delivery of family planning and malaria treatment). At the global level, our findings contribute to documentation of service disruptions related to a significant pandemic, and provides a model of how routine health information system data can be combined with modeling tools to provide robust, granular estimates of service disruption and associated mortality.

## Data availability statement

The data analyzed in this study is subject to the following licenses/restrictions: The data belongs to the Ministry of Helth of Mozambique. Data can be obtained upon request. Requests to access these datasets should be directed to QF, ferq09@gmail.com.

## Author contributions

OA, QF, KS, and BW conceptualized the research question, the study design, and the analytic strategy. QF, SC, and IM procured and secured the data. OA and TR performed the analysis with substantial inputs from BW and KS. OA developed the first draft with inputs from KS, TR, and BW. All authors provided substantial input, reviewed, and approved the final version.
