# Supplementary material for: Early effects of COVID-19 on maternal and child health service disruption in Mozambique
Source: Front Public Health. 2023 Apr 17;11:1075691. doi: 10.3389/fpubh.2023.1075691 (PMC10149948; doi:10.3389/fpubh.2023.1075691)

Table S1. Counts of missing data for completeness assessment

|  | **Jan 2017 - May 2020** | | **Jan 2018 - May 2020** | |
| --- | --- | --- | --- | --- |
|  | **Expected Obs** | **Misisng (%)** | **Expected Obs** | **Misisng (%)** |
| SMI.CPN...Mulheres.grávidas.que.receberam.a.3ª.dose..ou.mais..Sal.ferroso.com.Ac.Fólico | 6683 | 1.45 | 4727 | 1.44 |
| SMI.CPN...Mulheres.grávidas.que.receberam.1ª.Dose.VAT | 6683 | 1.72 | 4727 | 1.67 |
| SMI.CPN...Mulheres.grávidas.que.receberam.2ª.5ª.dose.VAT | 6683 | 1.41 | 4727 | 1.44 |
| SMI.CPN...Mulheres.grávidas.que.receberam.2.doses.de.TIP | 6683 | 1.47 | 4727 | 1.48 |
| SMI.CPN...Mulheres.grávidas.que.receberam.4.ou.mais.doses.de.TIP | 6683 | 1.54 | 4727 | 1.48 |
| SMI.CPN...mulheres.grávidas.que.fizeram.a.4.ou.mais.consultas.pre.natais | 6683 | 1.47 | 4727 | 1.46 |
| SMI.CPN...Mulheres.grávidas | 6683 | 1.36 | 4727 | 1.44 |
| SMI.MAT...Eclâmpsia | 6683 | 51.79 | 4727 | 52.55 |
| SMI.MAT...Administração.de.Anti.Hipertensivos | 6683 | 42.38 | 4727 | 42.33 |
| SMI.MAT...Antibióticos.parenterais.endovenosos | 6683 | 29.67 | 4727 | 30.63 |
| SMI.MAT...Hemorragia.pós.parto...Por.Atonia.uterina | 6683 | 68.04 | 4727 | 68.75 |
| SMI.MAT...Hemorragia.pós.parto...Por.Lacerações.do.Colo.e.Vagina | 6683 | 69.74 | 4727 | 69.85 |
| SMI.MAT...Pré.eclâmpsia.Severa | 6683 | 48.59 | 4727 | 48.00 |
| SMI.MAT...RN.com.Sépsis.Neonatal | 6683 | 85.47 | 4727 | 85.72 |
| SMI.MAT...RN.com.Asfixia.Grave | 6683 | 14.66 | 4727 | 14.75 |
| SMI.MAT...Reanimação.Neonatal | 6683 | 10.89 | 4727 | 11.57 |
| SMI.MAT...Recém.Nascido.em.contacto.imediato.pele.a.pele.com.a.mãe | 6683 | 1.38 | 4727 | 1.48 |
| SMI.MAT...Recém.nascido.amamentado.na.1ª.hora.após.o.parto | 6683 | 1.42 | 4727 | 1.52 |
| SMI.MAT...Total.de.Partos.na.Maternidade | 6683 | 1.30 | 4727 | 1.44 |
| VN...1ªs.Consultas | 6683 | 29.63 | 4727 | 41.44 |
| VN...Control.de.crescimento | 6683 | 29.90 | 4727 | 41.82 |
| MZ.SMI.CCS...Número.de.Crianças.12.59.Meses.que.receberam.o.Desparasitante | 6683 | 59.82 | 4727 | 43.20 |
| MZ.SMI.CCS...Número.de.Crianças.6.23.Meses.que.receberam.a.dose.de.MNP | 6683 | 72.42 | 4727 | 61.01 |
| MZ.SMI.CCS...Número.de.Crianças.com.Crescimento.Insuficiente.Atendidas | 6683 | 61.41 | 4727 | 45.44 |
| MZ.SMI.CCS...Número.de.Crianças.com.Desnutrição.Aguda.Atendidas | 6683 | 60.50 | 4727 | 44.15 |
| MZ.SMI.CCS...Número.de.Crianças.com.Desnutrição.cronica.Atendidas | 6683 | 66.05 | 4727 | 52.00 |
| MZ.SMI.CCS...Número.de.Crianças.de.Baixo.Peso.Idade.Atendidas | 6683 | 61.45 | 4727 | 45.50 |
| MZ.SMI.CCD...Nº.de.crianças.12.59.meses.que.receberam.o.desparasitante | 6683 | 60.93 | 4727 | 44.76 |
| MZ.SMI.CCD...Nº.de.crianças.suplementadas.com.vitamina.A | 6683 | 61.23 | 4727 | 45.19 |
| MZ.SMI.CCD...Primeiras.Consultas | 6683 | 60.00 | 4727 | 43.45 |
| MZ.SMI.CCD...Total.de.crianças.com.anemia.que.iniciaram.tratamento.com.sal.ferroso | 6683 | 62.76 | 4727 | 47.35 |
| MZ.SMI.CCD...Total.de.crianças.de.0.59.Meses.diagnosticadas.como.novo.caso.de..Pneumonia | 6683 | 60.59 | 4727 | 44.28 |
| MZ.SMI.CCD...Total.de.crianças.de.0.59.Meses.diagnosticadas.como.novo.caso.de.Diarreia | 6683 | 60.38 | 4727 | 43.98 |
| MZ.SMI.CCD...Total.de.crianças.de.0.59.Meses.diagnosticadas.como.novo.caso.de.Diarreia.e.tratadas.com.SRO.e.ZINCO | 6683 | 61.26 | 4727 | 45.23 |
| MZ.SMI.CCD...Total.de.crianças.de.0.59.Meses.diagnosticadas.como.novo.caso.de.Diarreia.e.tratadas.só.com.SRO | 6683 | 61.81 | 4727 | 46.01 |
| MZ.SMI.CCD...Total.de.crianças.de.0.59.Meses.diagnosticadas.como.novo.caso.de.Diarreia.e.tratadas.só.com.ZINCO | 6683 | 66.12 | 4727 | 52.10 |
| MZ.SMI.CCD...Total.de.crianças.de.5.14.diagnosticadas.como.novo.caso.de..Pneumonia | 6683 | 61.23 | 4727 | 45.19 |
| MZ.SMI.CCD...Total.de.crianças.de.5.14A.diagnosticadas.como.novo.caso.de.Diarreia | 6683 | 61.35 | 4727 | 45.36 |
| MZ.SMI.CCD...Total.de.crianças.de.5.14A.diagnosticadas.como.novo.caso.de.Diarreia.e.tratadas.com.SRO.e.ZINCO | 6683 | 64.03 | 4727 | 49.14 |
| MZ.SMI.CCD...Total.de.crianças.de.5.14A.diagnosticadas.como.novo.caso.de.Diarreia.e.tratadas.só.com.ZINCO | 6683 | 71.30 | 4727 | 59.42 |
| MZ.SMI.CCD...Total.de.crianças.observadas.com.baixo.peso.idade.grave | 6683 | 67.59 | 4727 | 54.18 |
| MZ.SMI.CCD...Total.de.crianças.observadas.com.baixo.peso.idade.moderado | 6683 | 63.98 | 4727 | 49.08 |
| MZ.SMI.CCD...Total.de.crianças.observadas.com.desnutrição.aguda.grave | 6683 | 66.18 | 4727 | 52.19 |
| MZ.SMI.CCD...Total.de.crianças.observadas.com.desnutrição.aguda.moderada | 6683 | 63.21 | 4727 | 47.98 |
| MZ.SMI.CCD...Total.de.crianças.de.0.59.meses.com.diagnostico.de.novo.caso.de.malaria.confirmada.e.tratadas.com.AL..Coartem. | 6683 | 60.39 | 4727 | 44.00 |
| MZ.SMI.CCD...Total.de.crianças.de.0.59.meses.com.diagnostico.de.novo.caso.de.malaria.confirmada.e.tratadas.com.Artesunato..AS. | 6683 | 68.82 | 4727 | 55.91 |
| MZ.SMI.CCD...Total.de.crianças.de.0.59.meses.com.diagnostico.de.novo.caso.de.malaria.confirmada.e.tratadas.com.Quinino | 6683 | 66.72 | 4727 | 52.95 |
| MZ.SMI.CCD...Total.de.crianças.de.0.59M.com.resultado.do.Teste.de.Malária.Positivo..TDR.ou.HTZ. | 6683 | 60.41 | 4727 | 44.02 |
| MZ.SMI.CCD...Total.de.crianças.de.5.14.anos.com.diagnostico.de..novo.caso.de.malaria.confirmada.e.tratadas.com.AL..Coartem. | 6683 | 60.60 | 4727 | 44.30 |
| MZ.SMI.CCD...Total.de.crianças.de.5.14.anos.com.diagnostico.de.novo.caso.de.malaria.confirmada.e.tratadas.com.Artesunato..AS. | 6683 | 72.63 | 4727 | 61.31 |
| MZ.SMI.CCS...Número.de.Crianças.Suplementadas.com..Vitamina.A | 6683 | 59.81 | 4727 | 43.18 |
| MZ.SMI.CCS...Primeiras.Consultas | 6683 | 59.81 | 4727 | 43.18 |
| SMI.MAT...Gravidez.Ectópica | 6683 | 86.11 | 4727 | 86.04 |
| SMI.MAT...Hemorragia.pós.parto...Por.Retenção.da.Placenta.ou.de.Restos.Placentares | 6683 | 46.09 | 4727 | 47.85 |
| SMI.MAT...Hemorragia.ante.parto...Placenta.Prévia | 6683 | 62.67 | 4727 | 62.22 |
| SMI.MAT...Malária | 6683 | 56.41 | 4727 | 58.01 |
| SMI.MAT...Número.de.Mulheres.que.fizeram.a.4ª.DOSE.ou.MAIS.de.TIP.na.Maternidade | 6683 | 37.60 | 4727 | 38.18 |
| SMI.CPP...Até.ao.2º.dia.após.o.Parto | 6683 | 1.39 | 4727 | 1.52 |
| SMI.CPP...Entre.o.3.º...7º.dias.após.o.Parto | 6683 | 8.56 | 4727 | 9.65 |
| SMI.CPP...Puérperas.com.Anemia | 6683 | 71.69 | 4727 | 73.81 |
|  |  |  |  |  |

Table S2a. Mortality estimates

|  | **Total expected deaths in 2020 with no disruptions (counterfactual)** | **Total estimated deaths in 2020 with observed disruptions** | **Additional deaths in 2020 due to service disruptions** | **Relative increase in mortality due to service disruptions** |
| --- | --- | --- | --- | --- |
| Child deaths (0-59 months) | 88,853 | 100,190 | 11,337 | 12.8% |
| Neonatal deaths (<1 month) | 50,311 | 56,016 | 5,705 | 11.3% |
| Maternal deaths | 5,084 | 5,471 | 387 | 7.6% |

S2b.

| **Child interventions** | **Additional child deaths**  **(0-59 months)** | **Relative contribution** |
| --- | --- | --- |
| Oral rehydration solution | 3,912 | 34.7% |
| Oral antibiotics for pneumonia | 1,188 | 10.6% |
| Case management of neonatal sepsis or pneumonia | 1,131 | 10.5% |
| Vitamin A for treatment of measles | 802 | 6.8% |
| Clean cord care | 705 | 6.4% |

S2c

| **Maternal interventions** | **Additional maternal deaths** | **Relative contribution** |
| --- | --- | --- |
| Parenteral administration of uterotonics | 174 | 44.9% |
| Parenteral administration of anti-convulsants | 56 | 14.5% |
| TT - Tetanus toxoid vaccination | 44 | 11.3% |
| Removal of retained products of conception | 33 | 8.5% |
| Assisted vaginal delivery | 22 | 5.6% |

**Additional Figures**

Figure S1. Number of women who attended first antenatal care visit


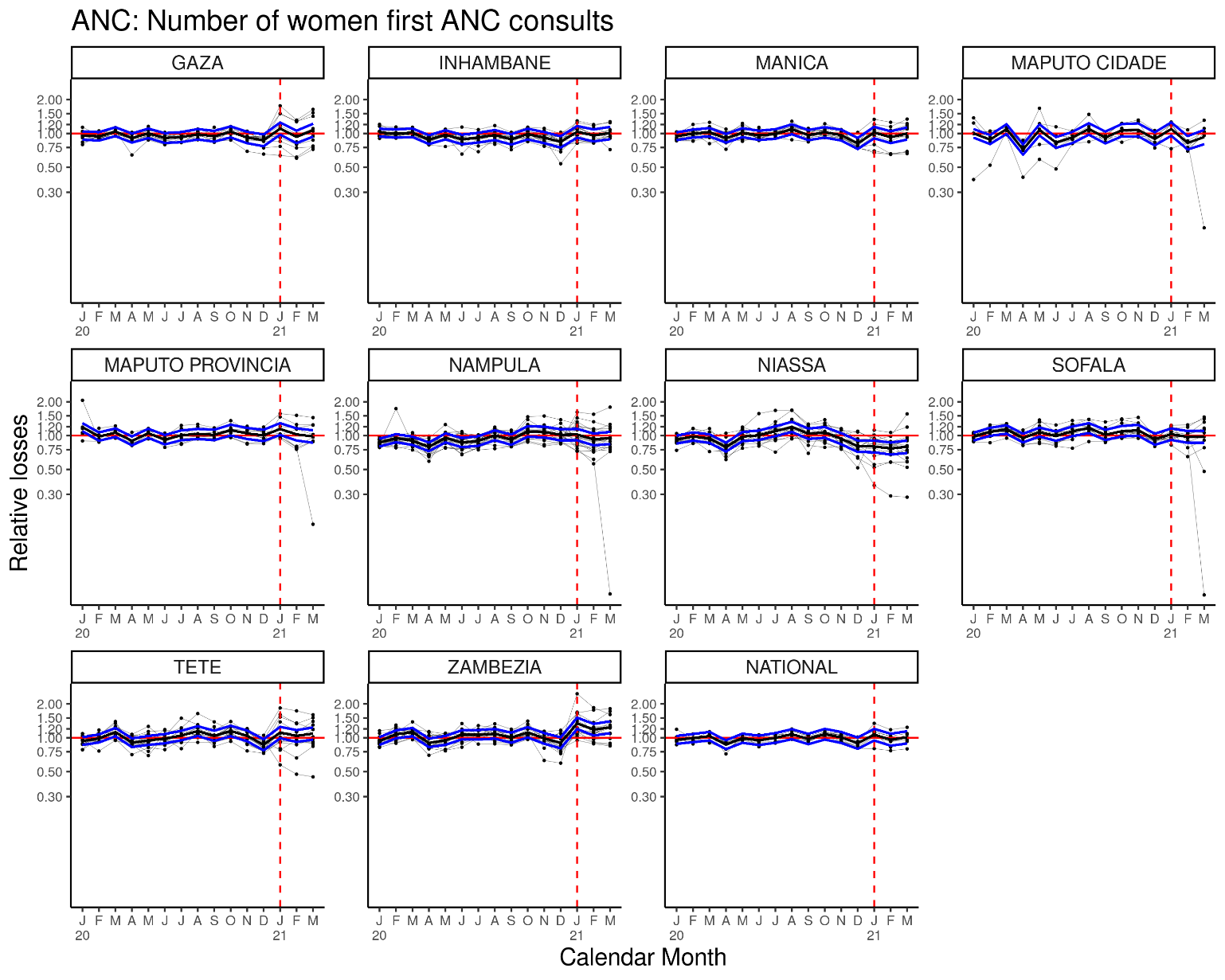


Figure S2. Number of women who gave birth in a facility


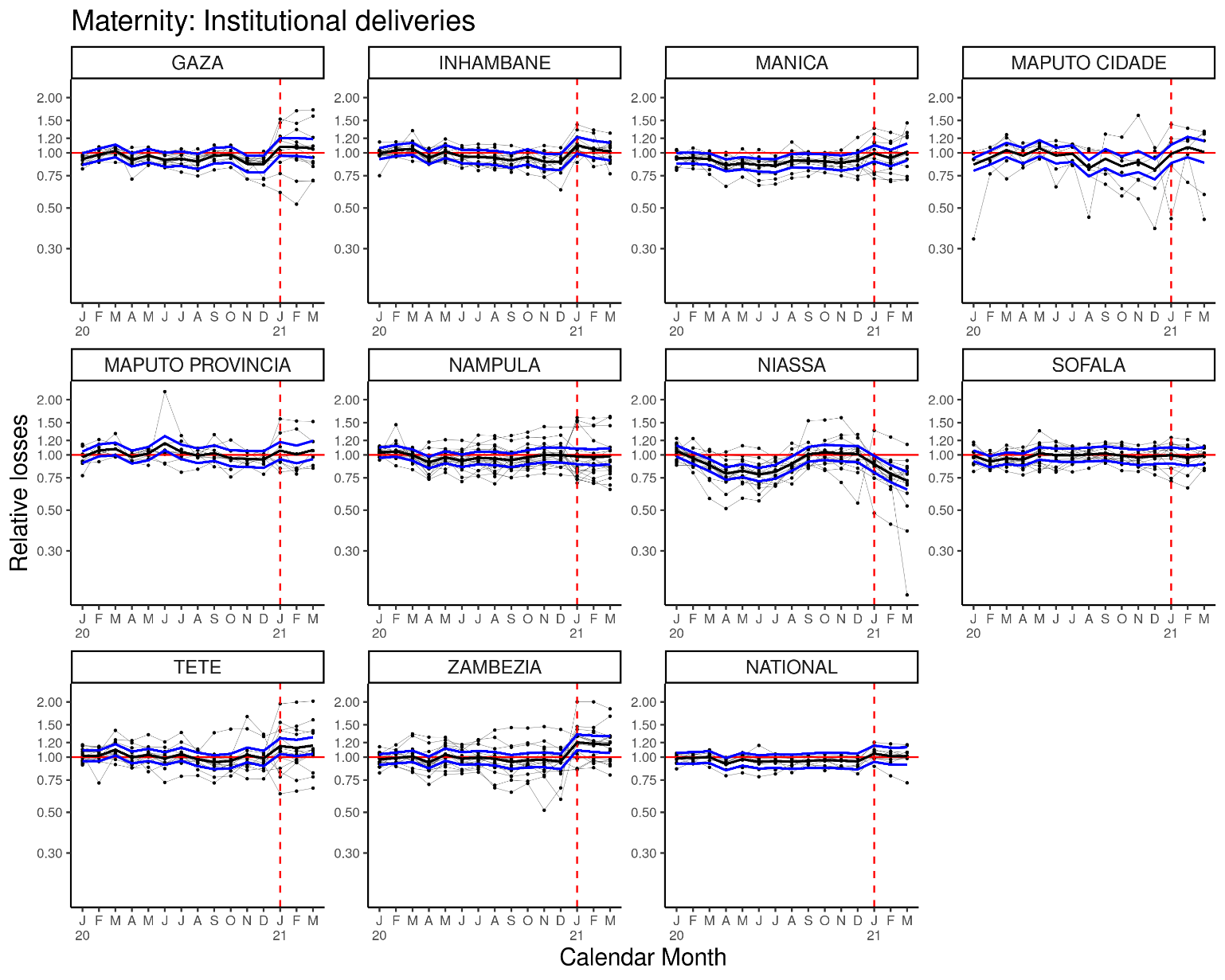


Figure S3. Number of visits for measles vaccination


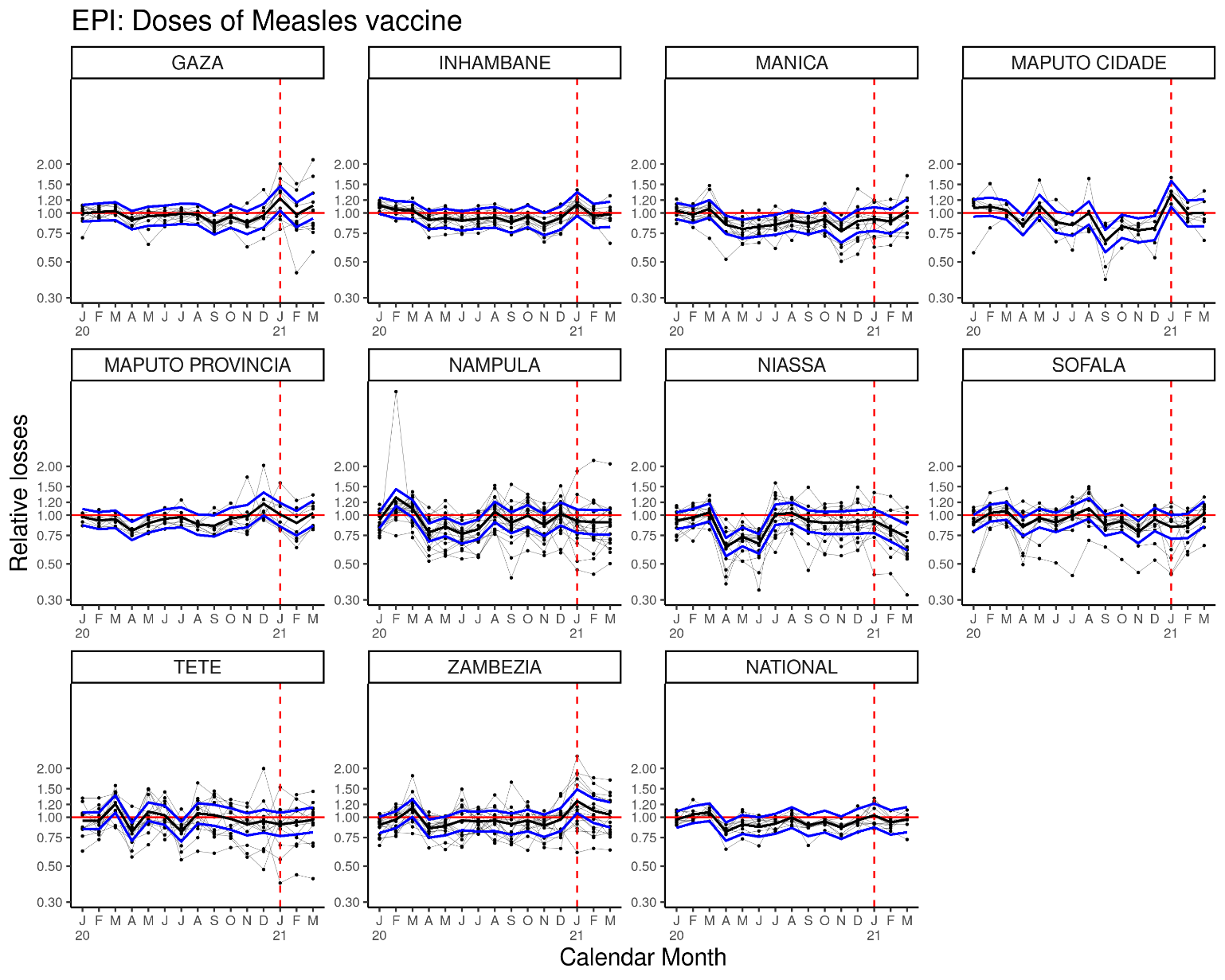


Figure S4. Number of visits for third dose of combined vaccine for diphtheria, pertussis, tetanus, and haemophilus influenza


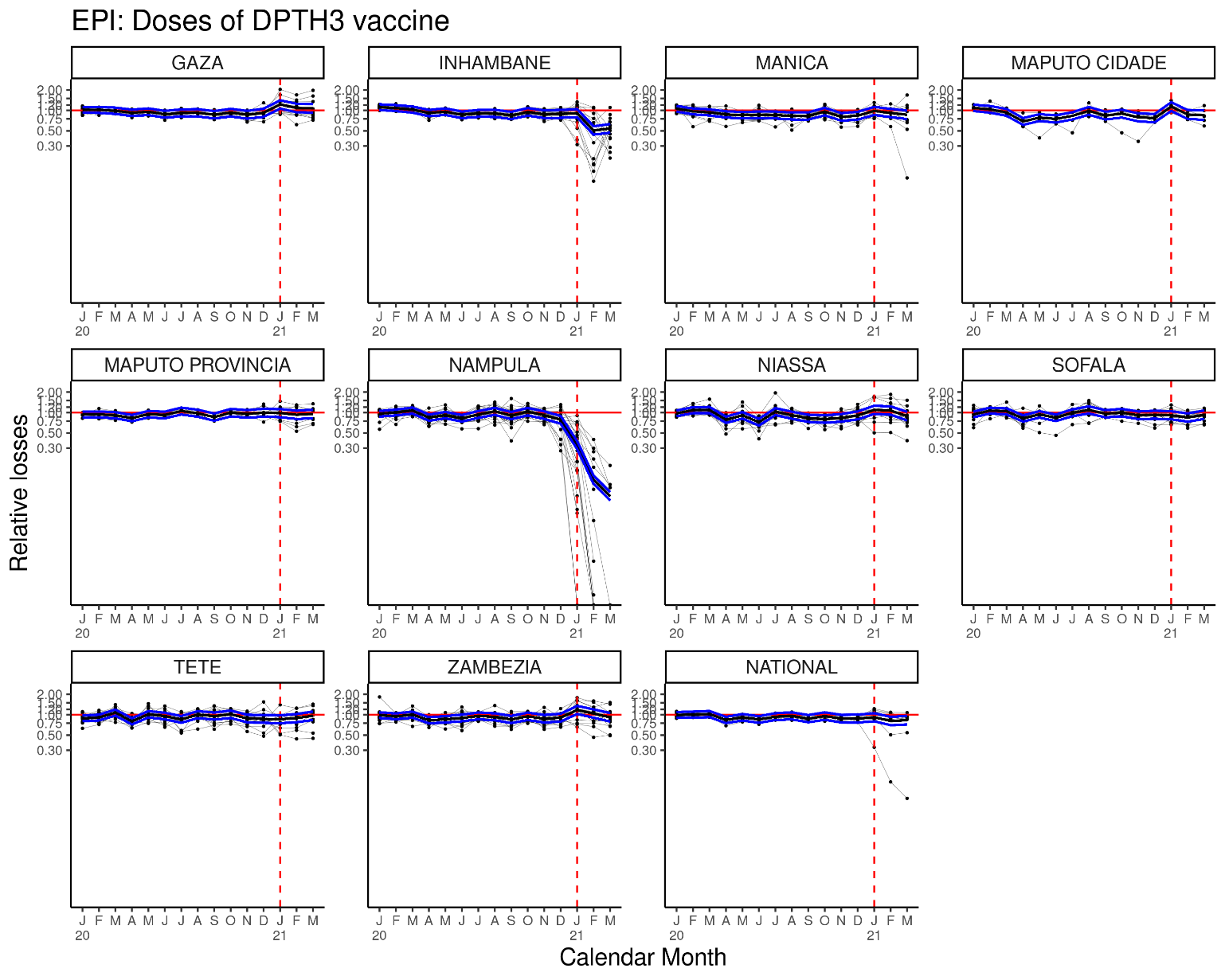


Figure S5. Number of postnatal care visits (within 48 hours after delivery)


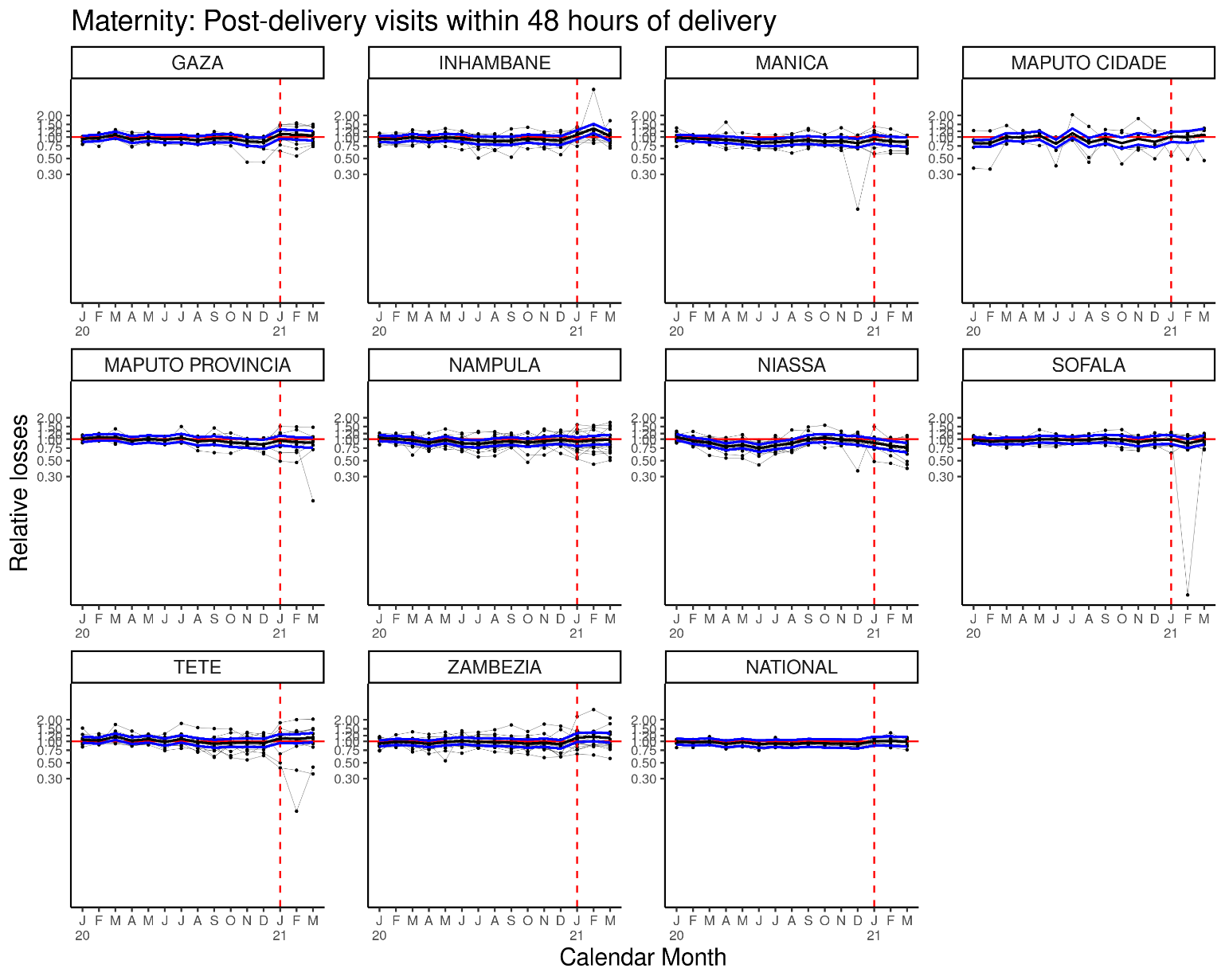


Figure S6. Number of postnatal care visits (within three to seven days after delivery)


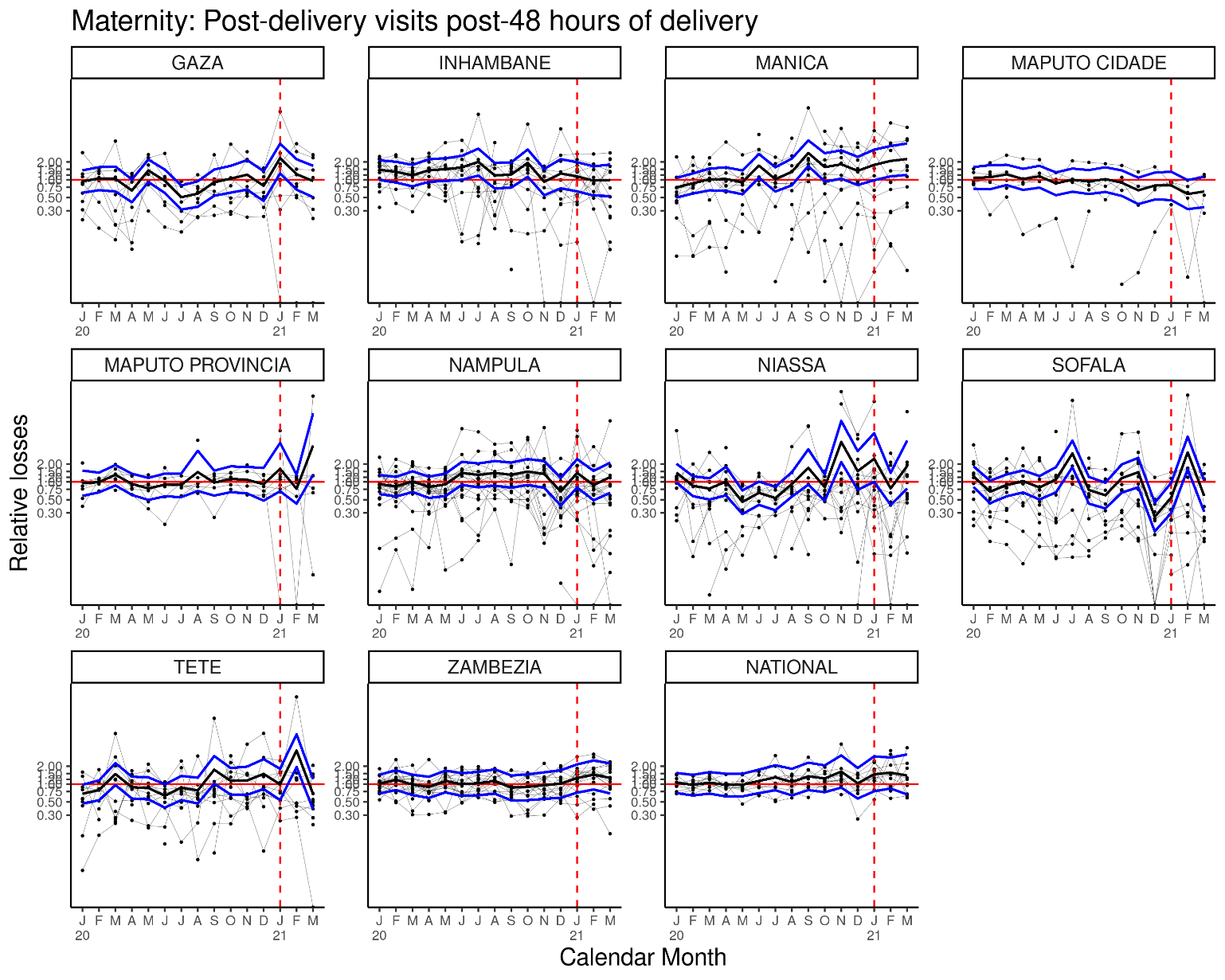


Figure S7. Number of children under five treated for malaria with Coartem


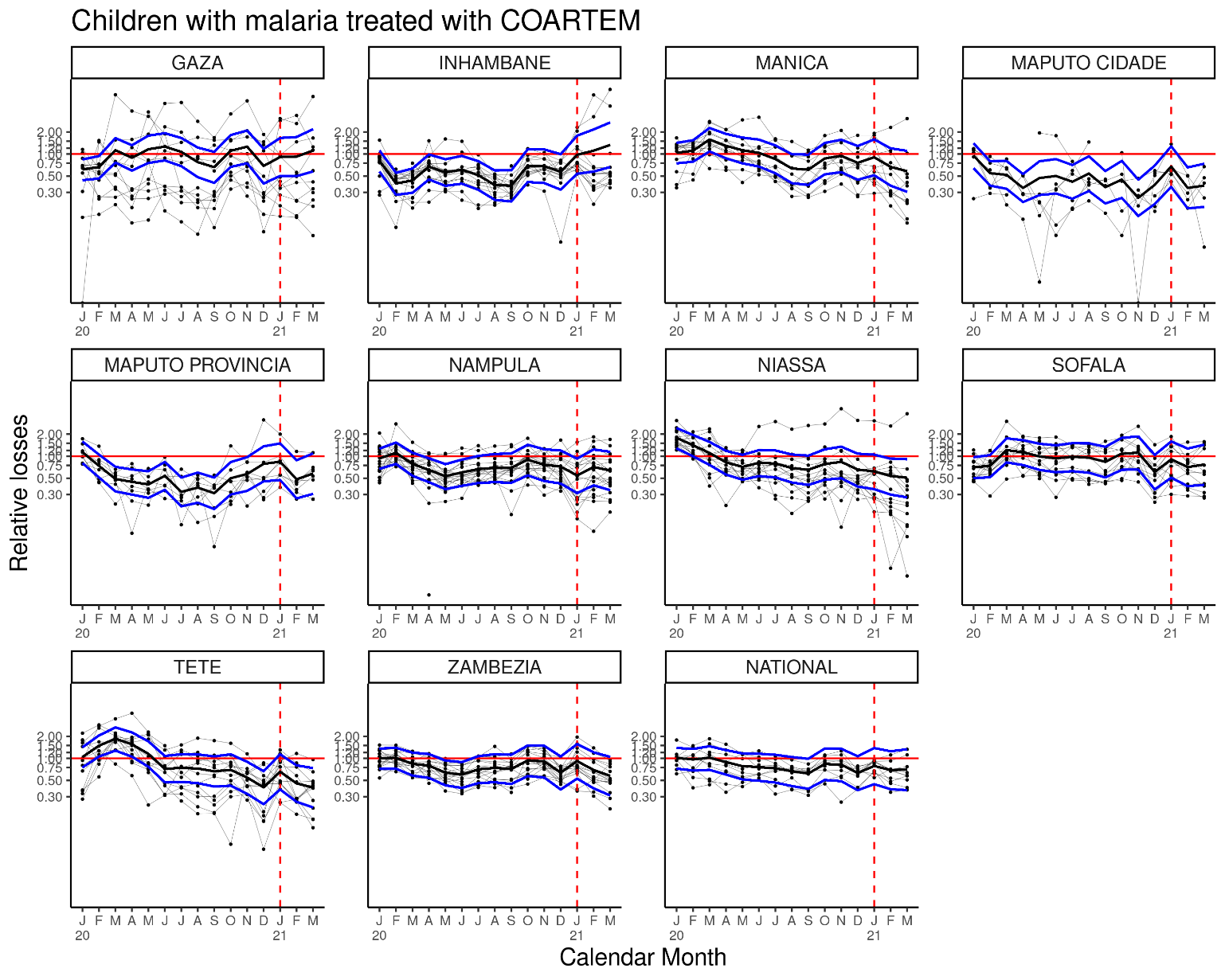


Figure S8. Number of first well-child visits


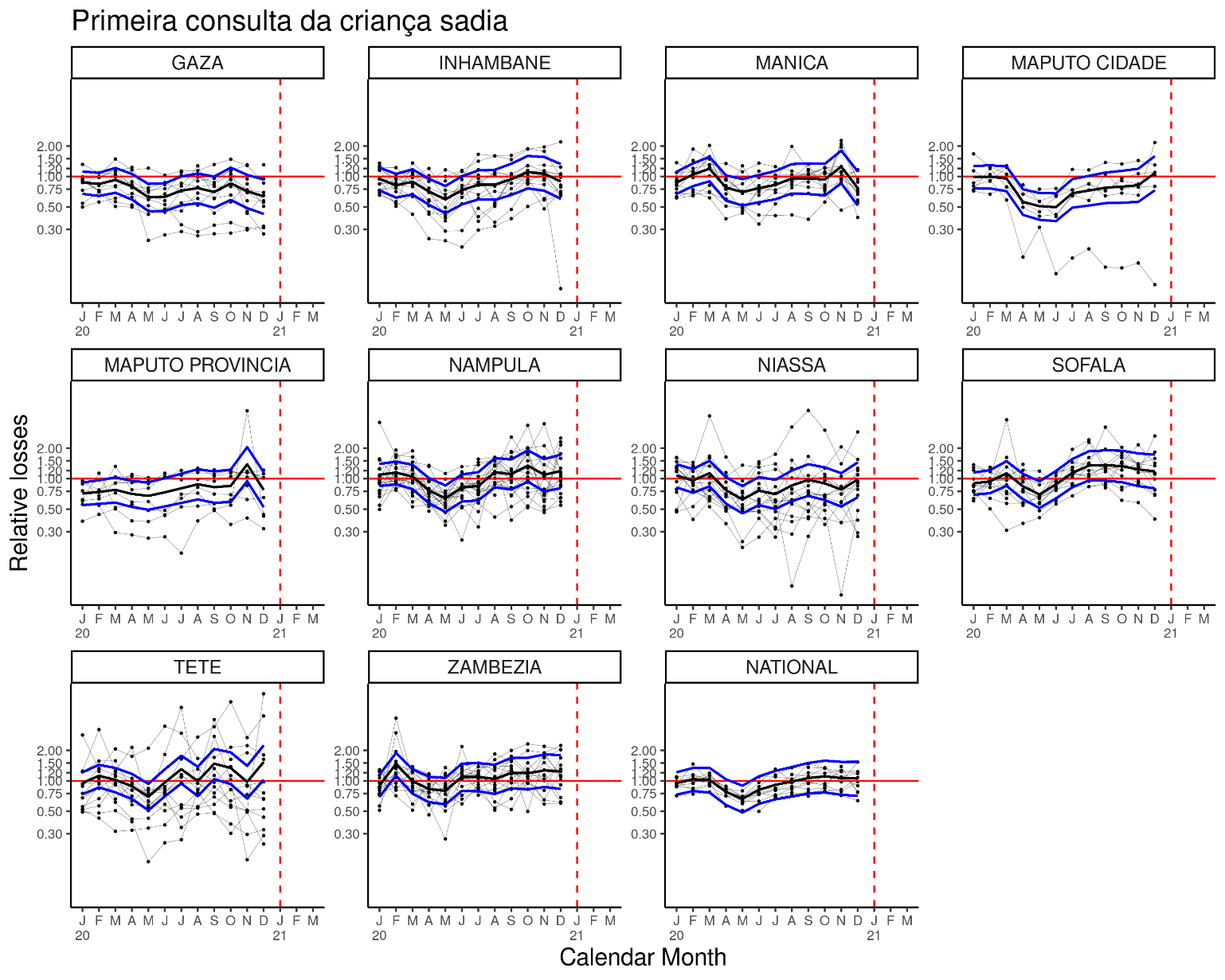


Figure S9. Number of family planning new users


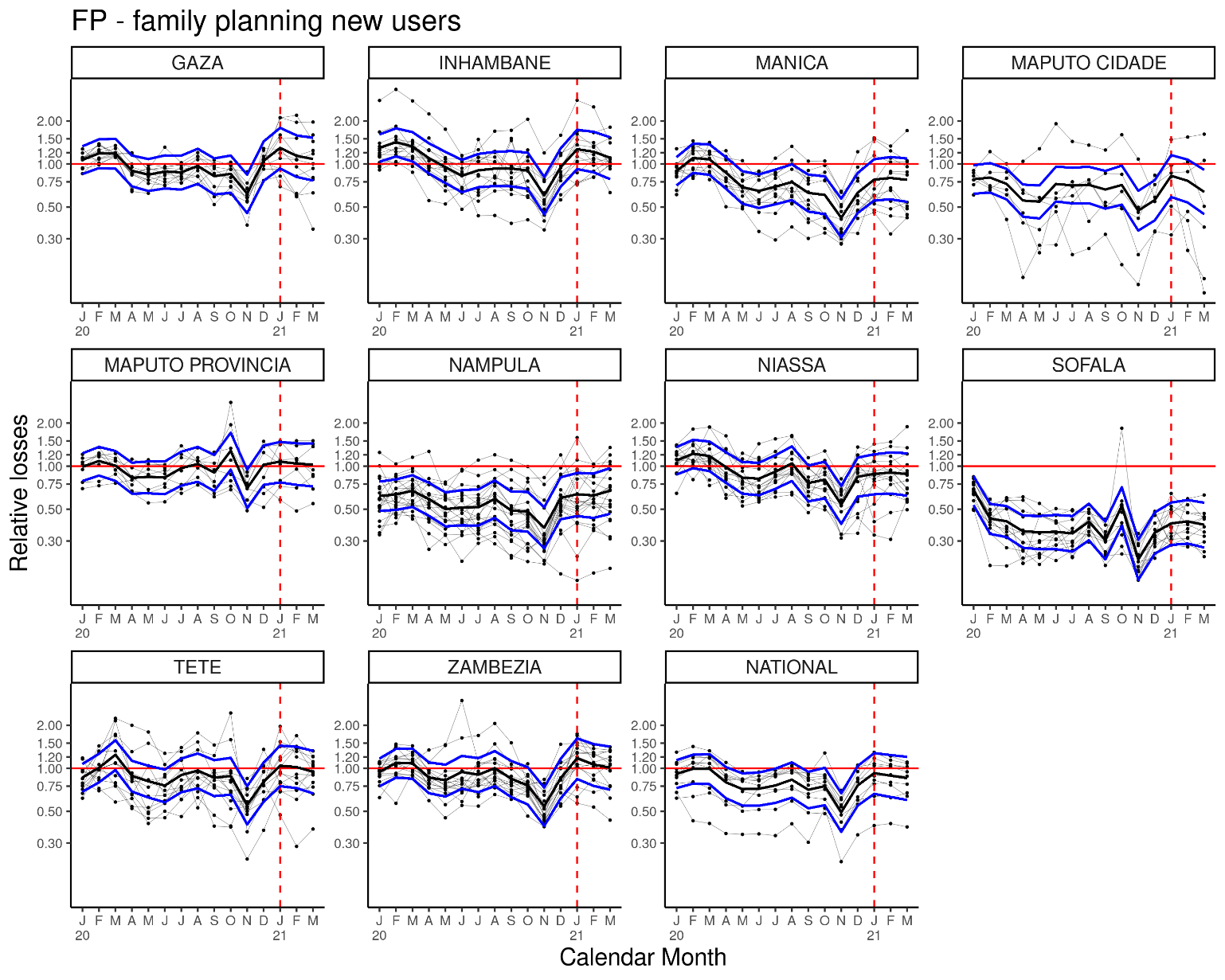

Supplement: Supplementary file 1 [file Data_Sheet_1.docx]
